# No More False Alert: Contrastive Learning for Predicting Health Deterioration from Imbalanced Care Records

**DOI:** 10.3390/s26113561

**Published:** 2026-06-03

**Authors:** Haru Kaneko, Sozo Inoue

**Affiliations:** 1 Department of Information Science, College of Humanities and Sciences, Nihon University, Sakura Jousui 3-25-40, Setagaya-ku, Tokyo 156-8550, Japan; 2 Department of Life Science and Systems Engineering, Kyushu Institute of Technology, 2-4 Hibikino, Wakamatsu-ku, Kitakyushu-shi 808-0196, Japan; sozo@sozolab.jp

**Keywords:** contrastive loss, care records, health deterioration prediction, imbalanced data

## Abstract

In this paper, we propose an outcome-based contrastive loss for imbalanced binary classification to alert to next-day health deterioration using care records and meteorological data. Long-term care facilities maintain daily care and observation records to monitor the health of older adults. Such objective records are particularly valuable when sudden deterioration occurs, enabling timely coordination with medical institutions. Predicting deterioration one day in advance could provide care staff with an actionable window to intensify observation and adjust care plans (e.g., scheduling additional vital checks or increasing fluid intake monitoring). This could potentially reduce emergency transports and ease the burden on already understaffed care facilities. However, for such predictions to be useful in practice, false positives must be suppressed. Because deterioration events are rare, class imbalance generates an excess of false positives, causing alert fatigue and increasing the risk that actual events go unnoticed. To address these challenges, we propose an outcome-based contrastive loss that contrasts actual deteriorating samples against false alarms conditioned on mini-batch prediction outcomes. The proposed loss contracts same-label pairs to shape local structure within each ground-truth label. The loss also separates actual deteriorating samples from false alarms among samples predicted as deteriorating, thereby directly reducing unnecessary alerts. As a result, compared with random oversampling with standard cross-entropy, the proposed model improved precision from 3.97% to 12.94% (+8.97 percentage points), while limiting the F1-score decrease to 0.71 percentage points (from 7.28% to 6.57%). Pair-design ablations and UMAP projections supported this mechanism by indicating clearer separation between actually deteriorating and false-alarm samples in the learned representation space. These results suggest a viable direction for alert systems that produce fewer unnecessary alerts, reducing alert fatigue and supporting more reliable deterioration detection in care settings.

## 1. Introduction

Population aging has substantially increased the number of older adults residing in long-term care facilities, placing mounting pressure on care staff who already face severe workforce shortages. In this setting, early detection of health deterioration is critical: if left unaddressed, deterioration can escalate to serious outcomes, including emergency transport and hospitalization. Long-term care facilities continuously accumulate daily care and observation records—covering vital signs, meal intake, excretion, and staff notes—that may capture early signals of health change. If deterioration can be predicted one day in advance from these records, care staff could potentially have an actionable window to intensify observation or adjust care plans before symptoms worsen. For example, staff could schedule additional vital sign checks or increase fluid intake monitoring. This could potentially reduce emergency transports and the burden on already understaffed facilities. A natural way to deliver such predictions to care staff is through an alert system that notifies them when the model detects a likely deterioration, potentially enabling earlier intervention.

However, a well-documented obstacle to deploying such alert systems is alert fatigue [1,2]. Frequent false alarms lead staff to distrust the system and disregard its alerts, a phenomenon also known as the cry-wolf effect. Once alert fatigue takes hold, actual deterioration events risk being overlooked, potentially negating the very benefit the system was designed to provide. The primary driver of alert fatigue is an excess of false positives (FPs). Health deterioration events are rare, creating a severe class imbalance. Such class imbalance is a pervasive challenge across health monitoring domains—from early warning scores in ICUs to chronic disease onset prediction—wherever clinical events are inherently rare [3,4,5]. This imbalance generates a flood of unnecessary alerts, imposing further burden on already overstretched care staff. Therefore, suppressing false positives while retaining true positives, that is, improving precision for the positive class, is not merely a performance objective. It is a prerequisite for practical deployment in real care settings.

In prior work [6], we attempted to predict health deterioration from care-record data. In that study, we found that strong class imbalance caused a large number of false positives, with negative-to-positive ratios reaching as high as 104:1. Excessive FPs lead to unnecessary alerts and alert fatigue, and this remains the central unresolved challenge that motivates the present work. Therefore, the alerting system requires FP reduction while retaining as many true positives (TP) as possible, and the objective prioritizes precision for the positive class.

For imbalanced data learning, data-level augmentation is often used, including random oversampling, random under-sampling [3], and SMOTE [7]. Imbalanced learning also uses algorithm-level designs, including class-weighted cross-entropy, focal loss [8], and threshold tuning. Many methods primarily improve minority-class detection and averaged metrics, such as the F1-score. These objectives do not explicitly encode the TP–FP separation inside the predicted-positive set. As a result, FP samples remain as confusing negatives near TP samples. In this setting, training may not sufficiently promote TP/FP separation, even when deployment prioritizes precision by suppressing FP. Contrastive loss directly controls distances between pairs of samples in the representation space, making it a natural fit for explicitly pushing TP and FP representations apart within the predicted-positive set—complementing cross-entropy in resolving the confusion patterns it leaves unaddressed.

In this paper, we propose an outcome-based contrastive loss for imbalanced binary classification to alert to next-day health deterioration from care records under severe class imbalance. Specifically, within each mini-batch, we assign samples to TP/FP/FN/TN based on model predictions. Based on this assignment, we define an outcome-based contrastive loss that contracts same–ground-truth-label pairs (TP–FN and TN–FP) and separates TP from FP within the predicted-positive set (i.e., samples predicted as positive). The overall objective loss is a weighted sum of cross-entropy and the proposed contrastive loss. The design explicitly promotes TP/FP separation within the predicted-positive set in the representation space. By mitigating confusion patterns that cross-entropy learning alone may not sufficiently resolve, we aim to suppress false positives (FP) while retaining true positives (TP).

As a result, compared with random oversampling with standard cross-entropy, the proposed method improved precision from 3.97% to 12.94% (+8.97 percentage points), while limiting the F1-score decrease to 0.71 percentage points (from 7.28% to 6.57%). The proposed method also outperforms the SMOTE baseline by 11.82 percentage points in precision and 4.35 percentage points in F1-score. These results support the proposed loss design, which suppresses false positives while retaining true positives. These findings suggest a viable direction toward alert systems that care staff can trust, enabling earlier intervention before health deterioration escalates in care settings.

## 2. Related Work

This section reviews related work on health deterioration prediction under severe class imbalance. In particular, we focus on false-positive (FP) suppression for alerting in imbalanced medical data.

### 2.1. Classification of Imbalanced Data

Random oversampling and random undersampling serve as standard remedies for class imbalance [3]. SMOTE and related methods generate synthetic samples from minority-class neighborhoods to augment data [7,9]. However, sampling or synthesis can amplify outliers and blur class boundaries by altering the training distribution, potentially leaving confusing negatives near the decision boundary and increasing FP risk [10].

At the algorithm level, class-weighted cross-entropy loss and focal loss increase learning pressure on the minority class. Focal loss [8] is a widely used baseline for severe class imbalance because it down-weights easy examples and emphasizes hard misclassified samples. However, these objectives are typically optimized for overall classification performance and do not explicitly encode operational preferences such as suppressing false alarms. As a result, the objective rarely specifies explicit separation between TP and FP inside the predicted-positive set. Cost-sensitive learning assigns misclassification costs to FN and FP and formulates training as expected-cost minimization [11,12,13]. However, many formulations emphasize decision-error weighting but do not directly specify the separation of the representation space for TP/FP confusion within the predicted-positive set. Many frameworks implement cost-sensitive prediction via probability estimation and cost-based decision rules with threshold selection [14,15]. Therefore, objectives rarely encode an explicit goal that directly reduces TP/FP confusion in the representation space inside the predicted-positive set.

Selective classification suppresses false alarms by abstaining from predictions on a subset of samples. SelectiveNet jointly controls coverage and selective risk [16]. However, abstention-based suppression differs from representation learning that targets TP/FP separation inside the predicted-positive set.

### 2.2. Predicting Health Condition Using Clinical Data and Care Records

Many studies in clinical medicine use structured electronic health record (EHR) data to predict deterioration and relapse. Such EHR data include vital signs, laboratory results, and medication histories. Moreover, EHR systems digitize clinical information and encode diagnoses with standardized identifiers. In this setting, existing studies have proposed EHR-based models for disease-risk prediction and chronic-disease onset prediction [17,18,19,20]. Representation learning further derives patient embeddings from EHR and extends risk prediction [21,22,23]. However, deterioration events often remain rare. Therefore, an improvement in AUROC alone does not ensure useful deployment. In practice, comparative evaluations including early warning scores (EWS) report low positive predictive value (PPV) [2,4,5,24].

Care facilities also maintain care records that document residents’ conditions and care activities, although research remains limited relative to clinical medicine. Care records continuously capture daily care and observed behaviors, even without laboratory tests, and care records resemble EHRs as structured health-related data. In contrast to EHRs, which mainly represent clinical encounters and treatments, “care records” mainly represent daily-life observations. Care records continuously log daily-life events such as vital signs, meal intake, excretion volume, bathing, going out, and rehabilitation. Existing studies predict excretion events from care-record system logs [25] and support automatic generation of care-record contents [26]. However, few studies predict health deterioration from care records and design learning objectives for alerting while suppressing false positives.

One study predicts health deterioration in older adults from self-reported data [27], building classification and regression models using lifestyle and environmental features and reporting strong classification performance for a deep neural network. Another predicts hospitalization among home- and community-based care users from administrative records [28]. However, self-reported frameworks rarely capture detailed daily care and observation signals recorded in care records. Moreover, low event prevalence often yields many false positives (FPs) in alerting, increasing workload, and alert fatigue. Therefore, we focus on a learning objective for next-day health deterioration prediction from care records and meteorological information, which suppresses false positives (FPs) while retaining true positives.

### 2.3. Contrastive Learning

Contrastive learning trains representations by directly controlling pairwise distances in a representation space [29,30,31,32]; SimCLR popularized this approach in self-supervised settings [33]. Supervised contrastive learning extends this framework to classification by constructing positive and negative pairs from class labels or auxiliary information. Representative methods include SupCon, which forms positive sets by class labels [34], and CLCE, which integrates cross-entropy with a label-based contrastive loss [35]. However, class imbalance can bias positive-set construction toward majority classes, and long-tailed extensions such as PaCo address this issue [36,37].

In a related direction, recent work defines positives and negatives based on model predictions and targets, thereby reducing misclassification risk. For selective classification, CCL-SC defines positives and negatives using prediction outcomes and confidence scores [38]. CCL-SC pulls together correctly classified same-class samples and pushes away samples misclassified into the same class to reduce selective risk. These supervised contrastive methods shape a representation space using labels or prediction correctness, and the present study shares this direction. However, to our knowledge, few studies explicitly distinguish prediction outcomes (TP/FP/FN/TN), design pair sets accordingly, and primarily optimize for false-positive (FP) suppression in a target class under severe imbalance.

### 2.4. Positioning of This Study

Existing imbalanced-learning methods (resampling, class weighting, and thresholding) mainly target minority-class detection and improvements in averaged metrics (e.g., F1-score and coverage–risk). These methods often fail to sufficiently suppress false positives (FPs) inside the predicted-positive set. FP samples often appear as hard negatives near true positives (TPs). We treat the issue as a form of confusion in the representation space. The proposed method introduces a contrastive loss that explicitly controls pairwise distances by prediction outcome (TP/FP/FN/TN). The loss pulls samples with identical ground-truth labels closer, and enforces a margin between TP and FP inside the predicted-positive set. In this way, the proposed method explicitly increases inter-class separation, thereby suppressing FP and improving precision.

## 3. Proposed Method

**Figure 1:**
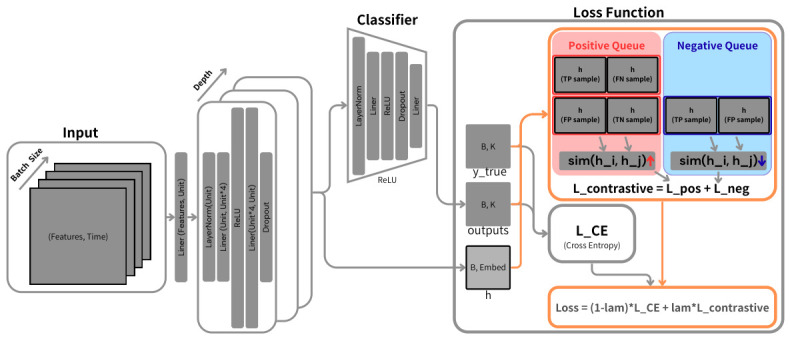
Overview of model architecture and loss computation in the proposed method. The model computes an intermediate representation from input features and jointly computes a cross-entropy-based classification output and a contrastive loss based on sample pairs inside each mini-batch.

This section defines the health deterioration prediction task using care records and then describes the prediction model and the loss function design (Figure 1). This section prioritizes improving precision for the positive class to suppress false positives (FPs), which drive alert fatigue. In this task, true positives (TP) and false positives (FP) often lie close inside the predicted-positive set. The proximity increases TP–FP confusion and triggers excessive alerts. Thus, the proposed method categorizes each mini-batch sample as TP/FP/FN/TN based on the prediction outcomes. The proposed method then explicitly defines sample-pair sets based on prediction outcomes. Specifically, the method minimizes cosine distance for same-label pairs (TP–FN and TN–FP). At the same time, the method enforces a margin on cosine distance for different-label pairs inside the predicted-positive set (TP–FP). This design directly promotes TP/FP separation in the representation space. The following sections formalize the pair sets and the loss function based on TP/FP/FN/TN.

We learn representations that separate TP samples from FP samples inside the predicted-positive set, in addition to the standard cross-entropy loss. We add a contrastive loss based on TP/FP/FN/TN types within each mini-batch and control the placement of misclassified samples in the representation space.

We define a mini-batch of size *n* as $B=\{x_{1},\ldots ,x_{n}\}$, and let [n]={1,…,n} denote the index set. For each sample $x_{i}\in B$ (i∈[n]), the ground-truth label is $y_{i}\in \left\{0,1\right\}$, the logit vector is $z_{i}=\left(z_{i,0},z_{i,1}\right)\in R^{2}$, the intermediate layer output is $h_{i}\in R^{d}$, and model $f_{\theta }$ outputs $\left(h_{i},z_{i}\right)$ for each *i*.

$$\left(h_{i},z_{i}\right)=f_{\theta }\left(x_{i}\right).$$

We define the predicted label as

$${\hat{y}}_{i}=\arg\max_{c\in \{0,1\}}z_{i,c}.$$

We set the positive (health deterioration) class as 1 and define index sets for TP, FN, TN, and FP in the mini-batch as

$$I_{\mathrm{TP}}=\left\{i\in \left[n\right]|y_{i}=1,{\hat{y}}_{i}=1\right\},$$

$$I_{\mathrm{FN}}=\left\{i\in \left[n\right]|y_{i}=1,{\hat{y}}_{i}=0\right\},$$

$$I_{\mathrm{TN}}=\left\{i\in \left[n\right]|y_{i}=0,{\hat{y}}_{i}=0\right\},$$

$$I_{\mathrm{FP}}=\left\{i\in \left[n\right]|y_{i}=0,{\hat{y}}_{i}=1\right\}$$

The loss formulation (cosine distance, margin, positive/negative pair objectives) follows a standard pairwise contrastive framework [29]. The contribution of this work lies in the pair-selection strategy. Unlike SupCon [34], which forms pairs by class labels, or CCL-SC [38], which uses prediction confidence, the proposed method defines pairs by TP/FP/FN/TN types. This design directly targets FP suppression within the predicted-positive set.

The contrastive term constructs positive pairs to pull together and negative pairs to push apart from these sets. The positive-pair set $P_{\mathrm{pos}}$ is defined as

$$P_{\mathrm{pos}}=P_{\mathrm{TP},\mathrm{FN}}\cup P_{\mathrm{TN},\mathrm{FP}},$$

$$P_{\mathrm{TP},\mathrm{FN}}\subset I_{\mathrm{TP}}\times I_{\mathrm{FN}},\;P_{\mathrm{TN},\mathrm{FP}}\subset I_{\mathrm{TN}}\times I_{\mathrm{FP}},$$

The negative-pair set $P_{\mathrm{neg}}$ is defined as

$$P_{\mathrm{neg}}=P_{\mathrm{TP},\mathrm{FP}},\;P_{\mathrm{TP},\mathrm{FP}}\subset I_{\mathrm{TP}}\times I_{\mathrm{FP}}$$

In implementation, each combination forms $\max(|I_{A}|,|I_{B}|)$ pairs, and random matching within the mini-batch fills missing pairs and constructs positive and negative pair sets *P*.

Next, the method computes the cosine distance $d_{ij}$ for each matched pair (i,j)∈P. The method applies $\ell_{2}$ normalization to each feature vector $h_{i}$ as

$${\tilde{h}}_{i}=\frac{h_{i}}{{∥h_{i}∥}_{2}}$$

and defines the cosine distance for pair (i,j) as

$$d_{ij}=1-{\tilde{h}}_{i}^{⊤}{\tilde{h}}_{j}$$

Given a margin m>0, the contrastive loss for positive and negative pairs is

$$L_{\mathrm{pos}}=\frac{1}{|P_{\mathrm{pos}}|}\sum_{\left(i,j\right)\in P_{\mathrm{pos}}}d_{ij}^{2},$$

$$L_{\mathrm{neg}}=\frac{1}{|P_{\mathrm{neg}}|}\sum_{\left(i,j\right)\in P_{\mathrm{neg}}}{\left[\max(0,m-d_{ij})\right]}^{2}$$

The method sets each term to 0 when the corresponding pair set is empty. The method defines the contrastive term as

$$L_{\mathrm{contrastive}}=L_{\mathrm{pos}}+L_{\mathrm{neg}}$$

and refers to this term as the contrastive loss. The cross-entropy loss is

$$L_{\mathrm{CE}}=\frac{1}{n}\sum_{i=1}^{n}\mathrm{CE}\left(z_{i},y_{i}\right)$$

The final loss *L* is

$$L=\left(1-\lambda \right)L_{\mathrm{CE}}+\lambda L_{\mathrm{contrastive}},\;0\leq \lambda \leq 1$$

Hyperparameter λ controls the contribution of the cross-entropy term and the contrastive term.

This pairing scheme pulls TP–FN and TN–FP as positive pairs and drives samples with identical ground-truth labels but different prediction outcomes toward similar representations, which facilitates the correction of misclassified samples beyond cross-entropy training. In parallel, the loss pushes TP–FP as negative pairs to keep distances above a margin and separates samples predicted as positive but associated with different ground-truth labels in the representation space, which strengthens TP/FP separation inside the predicted-positive set. This objective pushes confusing negative samples (FP) toward the negative side at the representation level while preserving correct positive samples (TP) as much as possible.

## 4. Experiment

**Figure 2:**
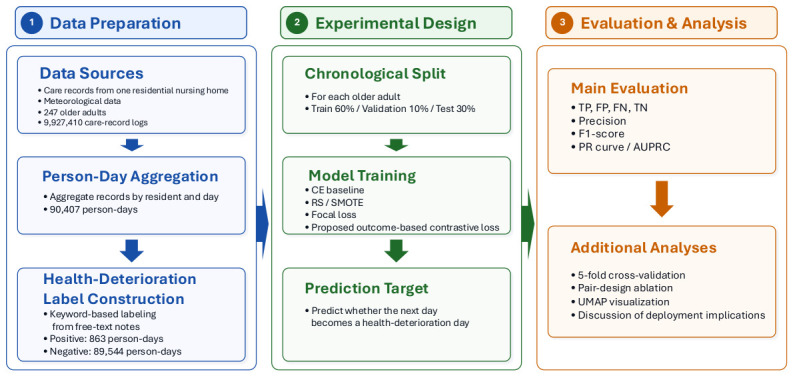
Overview of the study workflow across three phases. Phase 1 (Data Preparation) covers data collection, person-day aggregation, and keyword-based label construction. Phase 2 (Experimental Design) covers the chronological train/validation/test split and model training under multiple loss conditions. Phase 3 (Evaluation & Analysis) covers main performance metrics, 5-fold cross-validation, pair-design ablation, and UMAP visualization.

This section describes the experimental setup, using a health deterioration dataset comprising care records and meteorological data. First, this section reports the label frequency and symptom breakdown for health deterioration and clarifies severe class imbalance and operational constraints. Next, this section explains preprocessing and feature design, evaluation metrics, and baseline methods for comparison. Figure 2 provides an overview of the study workflow.

### 4.1. Data

This study uses a dataset that integrates care-record data routinely recorded in nursing facilities and meteorological data published by the Japan Meteorological Agency. Care records provide daily care contents and observation notes for each older adult, and meteorological data provide external environmental variables such as temperature and precipitation. The dataset aggregates information per person per day and formulates a binary classification that predicts whether the next day becomes a health deterioration day from information up to the current day.

#### 4.1.1. Data Detail

The care record data used in this study were collected at a residential nursing home in Japan and are provided for research by a care record system provider. To address privacy concerns, the care-record system provider supplied fully anonymized data after removing direct identifiers such as names and date of birth, and the dataset contains no information that enables personal identification. We use the data only for research purposes and manage the data under the ethics guidelines of our institution. The study obtains and uses meteorological data for the region of the facility from publicly available Japan Meteorological Agency datasets [39].

The care-record data used in this paper are collected from 247 older adults living in a residential nursing home in Japan between July 2021 and November 2023. All participants live inside the nursing facility, and daily living environments such as bathrooms and toilets are limited to on-site equipment. The facility provides three meals per day and offers snacks as needed, and staff measure vital signs routinely every morning.

Care staff enter these care records using the mobile application “FonLog” [40,41]. Each record includes the execution time, an older-adult ID, a care-action type, and action-specific details. The application records one care-action type via a selection form (e.g., meal, toileting, bathing, vital-sign measurement) and records action-specific detail items via selection forms, such as food intake, excretion volume, and stool condition. This study constructs a daily health deterioration label from symptom terms in free-text fields of care records. A day becomes a health deterioration day (positive; y=1) when free text includes at least one of the terms “Unwell”, “fever”, “abdominal pain”, “headache”, or “nausea”; otherwise, the day becomes a normal day (negative; y=0). Section 4.1.2 describes the label definition and statistical properties in detail.

#### 4.1.2. Data Characteristics

The care-record dataset includes 90,407 person-days and 9,927,410 records. The care-record system aggregates 112,230 activity instances. Observation days per older adult show a mean of 366.0±303.9 days (mean ± standard deviation) and a median of 232.0 days, ranging from 3 to 879 days. The number of daily records averages about 11 logs per day and shows large between-person variation (e.g., about 26 logs per day for high-frequency individuals). Some days contain zero logs because hospitalization or other reasons prevent record creation. This dataset represents an operational log with heterogeneous observation (recording) density.

**Figure 3:**
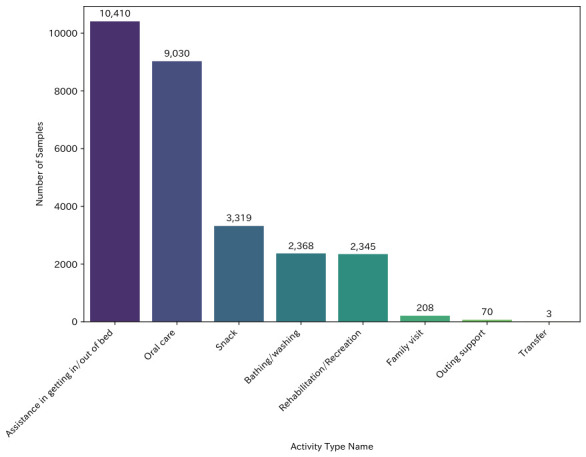
Record counts by all activity types. Recording frequency differs across activity types.

Figure 3 shows record counts by action type. Recording frequency varies substantially across action types, and a subset of categories accounts for a large share of records. Therefore, subsequent feature design requires daily aggregation that assumes category-level differences in recording frequency. Vital signs (e.g., body temperature and blood pressure) can appear multiple times per day, and this study uses daily averages when computing daily features.

**Figure 4:**
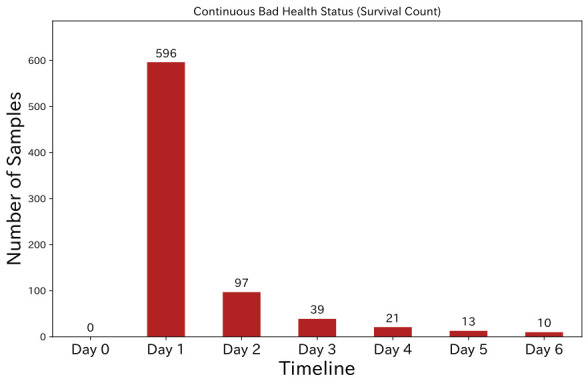
Count distribution of consecutive health-deterioration days (consecutive-day run length). The figure summarizes how long deterioration persists once it occurs, showing how often it appears as a single day versus continuous multi-days.

This section summarizes the keyword-defined “health deterioration day” label (y∈{0,1}). Among 90,407 person-days, 89,544 person-days are negative (normal day; y=0) and 863 person-days are positive (health deterioration day; y=1). The positive rate is about 0.95% (863/90,407), and the negative-to-positive ratio is about 104:1, which indicates severe class imbalance. Next, this section aggregates health deterioration frequency at the individual level. The analysis examines (i) the distribution of health deterioration days per person (median and IQR), (ii) the proportion of individuals with zero health deterioration days, and (iii) the distribution of consecutive health deterioration days (episode length). Figure 4 shows the count distribution by consecutive health deterioration days (episode length).

**Figure 5:**
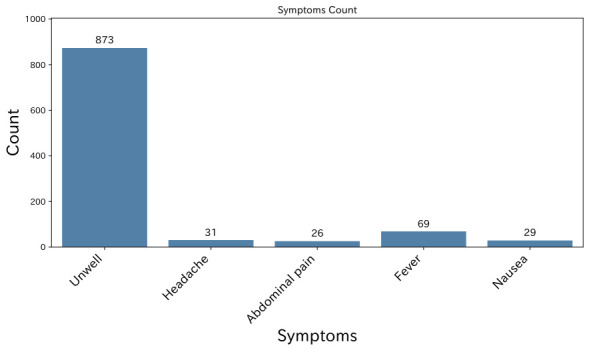
Occurrence counts of symptom terms (keywords) on health deterioration days.

The free-text fields on health deterioration days include multiple symptom terms. Figure 5 shows the counts of occurrences for each term of symptoms (keyword). This breakdown identifies symptom terms that mainly support the “health deterioration” label (e.g., dominance of a broad term such as “Unwell” or a high proportion of “fever”).

### 4.2. Preprocessing/Feature Calculation

Following prior work [6], the prediction task computes features from care records collected up to the previous day and predicts whether an older adult becomes a health deterioration case on the next day. Table 1 lists the features and computation procedures used in this paper. Feature extraction computes daily features for each older adult from care records and meteorological information. Meteorological variables (e.g., temperature and precipitation) come from Japan Meteorological Agency datasets [39]. The model does not use an older-adult identifier as a feature, which prevents any person-specific memorization.

Target label “health deterioration day” denotes a day whose free-text field in care records includes at least one of the terms “Unwell”, “fever”, “abdominal pain”, “headache”, or “nausea”. The health deterioration flag for the current day and the past deterioration counts in Table 1 are computed from information available up to and including the prediction day *t*. Because the task predicts health deterioration on the next day t+1, these variables are observed at prediction time and do not constitute label leakage. To prevent temporal leakage, the evaluation sorts data in chronological order for each older adult and uses the first 60% for training, the next 10% for validation, and the remaining 30% for testing. Because the split is strictly chronological, a resident whose stay spans the partition boundary will appear in both the training and test sets, but the test records always correspond to a later time period than the training records.

### 4.3. Evaluation Metrics

This study defines the positive class as a “health deterioration day” with label y=1. In an alerting setting, an increase in false positives (FP) triggers unnecessary responses and raises operational burden, so evaluation prioritizes precision for the positive class. All metrics below target the positive class (health deterioration) and do not use macro-averaging with negative-class metrics. Given predicted labels ${\hat{y}}_{i}\in \left\{0,1\right\}$ on the test set, counts of true positives (TP), false positives (FP), false negatives (FN), and true negatives (TN) are

$$\mathrm{TP}=\#\left\{i|y_{i}=1,{\hat{y}}_{i}=1\right\},\;\mathrm{FP}=\#\left\{i|y_{i}=0,{\hat{y}}_{i}=1\right\},$$

$$\mathrm{FN}=\#\left\{i|y_{i}=1,{\hat{y}}_{i}=0\right\},\;\mathrm{TN}=\#\left\{i|y_{i}=0,{\hat{y}}_{i}=0\right\}$$

Using these counts, the precision and F1-score for the positive class are

$$\mathrm{Precision}=\frac{\mathrm{TP}}{\mathrm{TP}+\mathrm{FP}}$$

$$F1=\frac{2\mathrm{TP}}{2\mathrm{TP}+\mathrm{FP}+\mathrm{FN}}$$

This study emphasizes higher precision by suppressing false positives (FP) while maintaining health deterioration detection performance (F1-score).

### 4.4. Baseline

To evaluate the proposed method, the experiments compare several network architectures. The experiments use a multilayer perceptron (MLP) with stacked fully connected layers and compare four configurations (Models 1–4) that vary only in hidden-layer width and depth. Each model uses ReLU activations in hidden layers and a two-class softmax output layer for binary classification. Baseline 1 trains these models using only class-weighted cross-entropy loss $L_{\mathrm{CE}(\mathrm{we})}$.

To compare with representative methods for imbalanced data, the experiments use random oversampling (RS), which randomly replicates minority-class samples, and SMOTE, which generates synthetic minority-class samples based on neighborhood information. Each method trains models under two loss settings: standard cross-entropy loss $L_{\mathrm{CE}}$ and class-weighted cross-entropy loss $L_{\mathrm{CE}(\mathrm{we})}$.

In addition, we include focal loss as an algorithm-level baseline for imbalanced learning [8]. We set γ=2 and apply the same class-weighting scheme as in $L_{\mathrm{CE}(\mathrm{we})}$ for the α term. This choice aligns the class-weighting scheme with $L_{\mathrm{CE}(\mathrm{we})}$, so differences mainly reflect the focal loss modulation of hard examples.

## 5. Results

This section reports experimental results for next-day health deterioration prediction from care records using the proposed method. First, this section compares network configurations trained with class-weighted cross-entropy loss and defines a baseline performance level for the task. Next, this section quantifies how the proposed contrastive objective suppresses false positives (FPs) while retaining true positives (TPs) relative to the baseline. Finally, this section analyzes TP/FP separation within the predicted-positive set and evaluates sensitivity to hyperparameters, including the margin parameter *m* and the weight parameter λ, to clarify design trade-offs.

### 5.1. Preliminary Investigation of Model Configurations

Table 2 shows baseline results for models trained only with class-weighted cross-entropy loss. All models share an identical block-structured fully connected network, and the comparison considered four configurations that vary only in hidden-layer width and the number of stacked blocks.

Models 3 and 4 achieved relatively higher F1-scores, and Model 4 attained the highest precision. Across all configurations, true positives for the positive class (health deterioration days) remained in the range of 6–12, and false negatives exceeded 200. The baseline yielded limited positive detection under severe class imbalance.

### 5.2. Comparison with Proposed Method and Existing Methods

This section compares prediction performance across standard class-imbalance baselines and the proposed method. This section fixes the network architecture to Model 3 to isolate the effect of each imbalance-handling strategy. The experiments evaluate random oversampling (RS) and SMOTE as baselines and optimize each under both the standard cross-entropy loss $L_{\mathrm{CE}}$ and the class-weighted cross-entropy loss $L_{\mathrm{CE}(\mathrm{we})}$. We also evaluated focal loss as a loss-based baseline for imbalanced learning. The proposed method sets λ=0.7 and m=0.3 and optimizes $L_{\mathrm{total}}=\left(1-\lambda \right)L_{\mathrm{CE}}+\lambda L_{\mathrm{cont}}$. Table 3 shows TP/FN/FP/TN counts together with precision and F1-score for each method.

**Figure A1:**
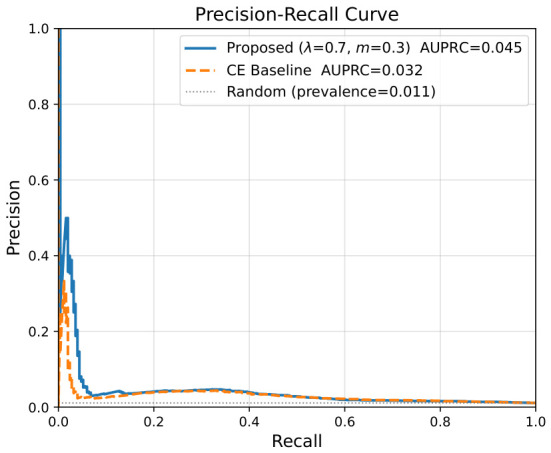
Precision-recall curves for the proposed method (λ=0.7, m=0.3) and the CE baseline. AUPRC =0.045 (proposed) versus 0.032 (CE baseline). The dotted line indicates random-classifier performance at class prevalence =0.011.

Compared with the class-weighted cross-entropy baseline (Table 2), the proposed method increased TP (7→11) and decreased FP (135→74). Precision increased to 12.94% (from 4.93%), and the F1-score increased to 6.57% (from 3.57%). Relative to this cross-entropy baseline, these concurrent gains indicated that the proposed method reduced false alarms without degrading detection performance. This behavior aligned with the operational goal of precision-oriented alerting. Notably, the proposed method achieved the highest precision among the compared baselines in Table 3. This result supported its suitability for practical deployment settings where false-alarm burden is critical. Figure A1 shows the precision-recall curves for the proposed method and the CE baseline, yielding AUPRC =0.045 versus 0.032, respectively. This suggests better overall precision–recall performance on the single-split test set and indicates that the observed precision advantage is not limited to the default threshold of 0.5.

RS and SMOTE increased TP but inflated FP, thereby reducing precision. For example, RS with $L_{CE(normal)}$ yielded an F1-score of 7.28%, but the setting produced many FPs. Similarly, SMOTE increased TP, but the setting generated many FPs. Therefore, the proposed method prioritized false-positive suppression more directly than resampling baselines and aligned with precision-oriented alerting. Focal loss increased TP but also substantially increased FP, which reduced precision. This result shows that focal loss favors detection-oriented operation but is less suited to alerting scenarios that prioritize reducing false-alarm burden in this dataset.

### 5.3. Ablation and Analysis

This section conducts analyses to better understand the behavior of the proposed contrastive loss. First, a 5-fold cross-validation assesses the robustness of the main results across different data partitions. Next, an ablation study switches pair definitions to examine contributions of each outcome-based pair (TP–FN, TN–FP, TP–FP, and FN–TN). Then, UMAP visualizes the intermediate representations and reveals how TP and FP placements differ between the baseline and the proposed method. Finally, performance under different values of the margin *m* and the loss weight λ is evaluated to assess sensitivity to these hyperparameters.

#### 5.3.1. Robustness Evaluation via Cross-Validation

To verify that the single-split results in Table 3 are not artifacts of a particular data partition, we conducted a 5-fold cross-validation using the same network architecture (Model 3) and hyperparameters (λ=0.7, m=0.3). The folds are split chronologically, so future information from later records does not leak into earlier training folds even when the same resident appears in multiple folds. Table 4 reports the mean and standard deviation of TP, FN, FP, TN, F1, and precision across folds.

The proposed method achieved the highest precision in all five folds (mean 9.47±8.09%). Notably, the mean FP count (319.2±97.4) was substantially lower than all baselines, which ranged from 332.8 (SMOTE, $L_{CE(normal)}$) to 11412.0 (RS, $L_{CE}$). These results confirm that the false-positive suppression effect of the proposed contrastive objective is robust across different data partitions. The large standard deviation of precision (8.09% relative to a mean of 9.47%) reflects the small number of positive samples per fold under severe class imbalance, and is consistent with the fold-level variance observed across all methods. Thus, while the cross-validation results support the relative false-positive suppression effect, they also indicate that absolute precision can vary substantially across temporal partitions and resident/cohort compositions. This variability may partly reflect heterogeneity in resident-level deterioration patterns and time-period-specific event composition. However, the present fold-level analysis does not isolate individual-level predictability, which should be examined in future resident-stratified analyses. Separately, regarding optimization stability, the validation loss of the proposed method converged smoothly and remained stable in the later epochs (epochs 50–100: mean = 0.120, std = 0.002), indicating that noisy pair assignments early in training did not destabilize the learning process.

#### 5.3.2. Ablation Study: Contribution Analysis of Pair Design

This section evaluated the impact of outcome-based pair definitions in the proposed contrastive loss on prediction performance. To isolate the contribution of pair design, we fixed Model 3 and set the margin m=0.3 and weight λ=0.7. Table 5 shows results obtained by switching the pair set used in $L_{\mathrm{cont}}$. The table covers all $2^{3}-1=7$ non-empty subsets of the three main pair types (TP–FN, TN–FP, TP–FP), including the full proposed method as the three-term subset. The FN–TN pair is included as a negative-control variant outside this framework.

Using only $L_{cont(\mathrm{TP}\text{-}\mathrm{FP})}$ improved precision from 4.21%→5.96% and increased the F1-score from 3.64%→4.49%, which indicated that the TP–FP separation term mitigates confusion inside the predicted-positive set. Among single-pair variants, $L_{cont(\mathrm{TN}\text{-}\mathrm{FP})}$ suppressed false positives to FP=55 and achieved a precision of 12.70%. However, this pair reduced true positives to TP=8 and limited the F1-score to 5.11%. In contrast, using only $L_{cont(\mathrm{TP}\text{-}\mathrm{FN})}$ yielded TP=12 and an F1-score of 6.05% while producing FP=135, which weakened false-positive suppression. $L_{cont(\mathrm{FN}\text{-}\mathrm{TN})}$ yielded TP=0 and failed to drive any positive detection. Therefore, TN–FP primarily supports false-positive suppression, and TP–FN primarily supports true-positive retention, so the two terms play complementary roles. Furthermore, the best setting in Table 3 (λ=0.7) combines positive-pair terms with the TP–FP negative-pair term and achieves higher precision by strengthening TP/FP separation within the predicted-positive set. Among the two-term combinations, TP–FN + TP–FP achieved F1=6.94% and Prec=12.50%, approaching the full method, whereas TN–FP + TP–FP reached F1=5.67% and Prec=9.71%. The full proposed method, combining all three terms, achieved the best balance of F1 and precision (F1=6.57%, Prec=12.94%), confirming that TN–FP and TP–FN play complementary roles in jointly suppressing false positives and retaining true positives.

#### 5.3.3. Visualization Analysis of the Representation Space (UMAP)

**Figure 6:**
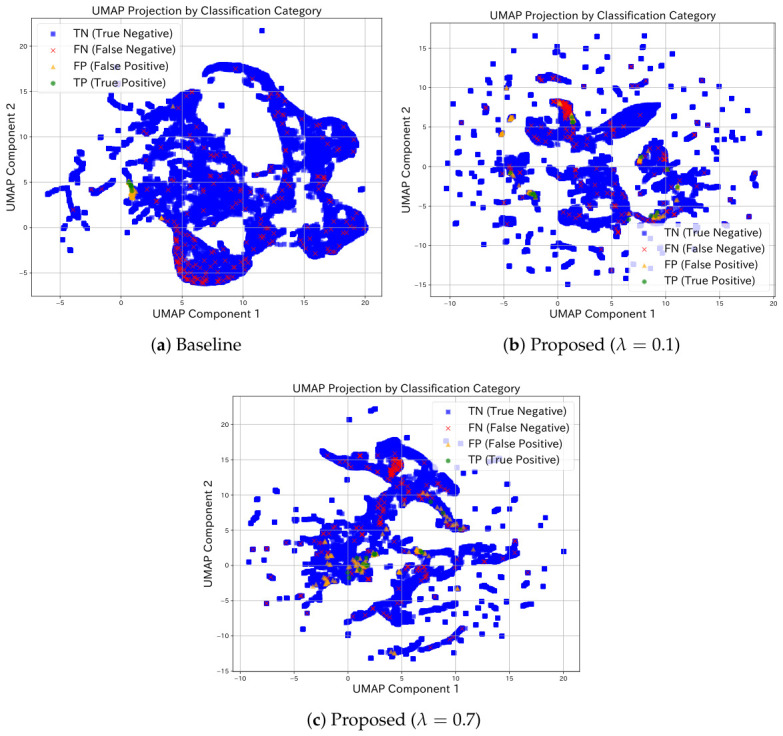
Representation-space comparison between the baseline and the proposed method. UMAP projected intermediate-layer outputs on the test set into two dimensions for models trained with Model 3. Subfigures show (**a**) the baseline trained with class-weighted cross-entropy loss, (**b**) the proposed method with λ=0.1 and m=0.3, and (**c**) the proposed method with λ=0.7 and m=0.3. The baseline concentrated true positives (TP) and false positives (FP) in nearly the same region, whereas the proposed method separated TP and FP more clearly, thereby improving TP/FP discrimination.

Figure 6 contrasts the representation spaces learned by the baseline and the proposed method. Each subfigure projected intermediate-layer representations from the test set in two dimensions with UMAP [42] and revealed the distribution by outcome category (TP/FP/FN/TN). The baseline corresponded to the Model 3 optimized with class-weighted cross-entropy loss alone (in Table 2). The proposed method is shown under two settings from Table 6 (λ=0.1 and λ=0.7, both with m=0.3) to visualize how the loss weight reshapes the geometry of the representation. All variants retained the Model 3 backbone and switched only the loss terms to preserve a controlled comparison.

In the baseline (Figure 6a), true positives (TP) and false positives (FP) clustered in a shared region, and the classifier labeled neighboring samples as positive and generated many false alarms. This overlap matched the baseline outcome, with FP=135 and a precision of 4.93%, indicating insufficient discrimination within the predicted-positive set. Prior work on contrastive learning links this type of predicted-positive crowding to cross-entropy objectives that weakly enforce inter-class margins and leave hard negatives near TP [35,38]. Such crowding arises because cross-entropy objectives prioritize decision-score separation and weaken constraints on representation geometry. As a result, embeddings tend to concentrate in a narrow band near the decision boundary.

In contrast, the proposed method spreads TP and FP across a wider region in both panels, thereby reducing local TP/FP entanglement. This reallocation reduced FP and improved the precision and F1-score. Therefore, the UMAP projections supported the view that the proposed loss strengthened TP/FP separation inside the predicted-positive set and improved precision-oriented alerting. We attempted to compute silhouette scores for TP vs. FP separation; however, with only 7–16 TP samples in the test set, the metric does not yield statistically reliable estimates. We therefore treat the UMAP projections as a qualitative illustration of how the representation structure changes under the proposed loss.

#### 5.3.4. Hyperparameter Sensitivity Analysis: Loss Weight Parameter “λ”

This subsection evaluated the sensitivity of the proposed method to the loss weight λ. We used Model 3, fixed the margin at m=0.3, and trained models with $L_{\mathrm{total}}=\left(1-\lambda \right)L_{\mathrm{CE}}+\lambda L_{\mathrm{cont}}$. Table 6 shows the prediction performance across λ.

Performance varied non-monotonically across λ, but moderate-to-high weights (λ=0.6–0.8) generally suppressed false positives and achieved higher precision than most lower-weight settings. However, larger λ did not monotonically improve overall performance, and overly large values reduced TP. Specifically, λ=0.7 achieved the highest F1-score (6.57%) with FP=74 and precision 12.94%, whereas λ=0.8 achieved the highest precision (16.28%) but reduced TP to 7 and F1-score to 4.78%. At λ=0.9, performance degraded substantially, with TP=5, precision 2.34%, and F1 2.16%. The F1-score peaked at λ=0.7 (6.57%), which provided the best trade-off between false-positive suppression and true-positive retention. Overall, λ∈[0.6,0.8] formed an effective search range on this dataset: λ=0.7 suited F1-oriented operating points, whereas λ=0.8 suited precision-oriented operating points.

#### 5.3.5. Hyperparameter Sensitivity Analysis: Margin “*m*”

This subsection evaluated the sensitivity of the proposed method to the negative-pair margin *m*. The experiments fixed Model 3, set the loss weight to λ=0.7, and varied only *m* to isolate its effect. Table 7 shows TP/FN/FP/TN counts together with precision and F1-score for each *m*.

With a small margin, weak TP–FP separation left residual confusion inside the predicted-positive set and increased false positives. In particular, m=0.1→0.3 yielded FP:160→74 and increased precision from 6.43%→12.94%. However, a larger *m* did not guarantee monotonic gains, and excessive separation pressure could conflict with the classification objective and harm true-positive retention. For example, m=0.3→0.7 increased FP from 74→124 and reduced precision from 12.94%→8.15%. Furthermore, m=0.3→1.0 reduced FP:74→67 but shifted TP:11→9 and decreased the F1-score from 6.57%→5.52%. Therefore, on this dataset, m=0.3 yielded the most favorable balance between precision and F1-score, and *m* primarily served as a tuning knob for adjusting the false-positive count.

## 6. Discussion

### 6.1. Results on Health Deterioration Prediction

We examined an outcome-based contrastive loss for next-day health deterioration prediction under severe class imbalance. The proposed method was designed to suppress false positives while preserving true positives for precision-oriented alerting. Compared with the class-weighted cross-entropy baseline for Model 3, the proposed method yielded TP:7→11 and FP:135→74 (Table 3). The shift was reflected in a precision increase from 4.93% to 12.94% and an F1-score increase from 3.57% to 6.57%, which was consistent with reduced confusion inside the predicted-positive set. These results confirm that precision improved substantially while the F1-score was also maintained, indicating that false-positive suppression was achieved without sacrificing true-positive detection. Reducing false positives by 45% (135 → 74) means fewer unnecessary alert responses for care staff, thereby directly lowering the risk of alert fatigue.

However, the best setting still achieved a precision of 12.94%, so the false-alarm burden would remain in deployment. The single-split evaluation in Table 3 remains the primary analysis for the intended accumulated-history setting and yielded a precision of 12.94%. However, this value should be interpreted as a point estimate for that setting, rather than as a fixed precision level expected under all deployment conditions. The 5-fold cross-validation (Table 4, 9.47% ± 8.09%) provides a complementary robustness analysis and indicates that absolute precision can vary substantially across temporal partitions and resident/cohort compositions. Therefore, the precision estimate from this single-split evaluation should be generalized cautiously to deployment settings with different temporal or resident/cohort compositions, although the proposed method consistently achieved the highest precision across all folds.

To quantify the operational alert burden, the proposed method generated 85 alerts (11 TP + 74 FP) over 22,742 test person-days, corresponding to approximately 3.7 alerts per 1000 resident-days. For a facility of 50 residents, this translates to roughly one alert every five days on average. Whether this alert frequency is operationally acceptable depends on staffing levels, workflow, and care context, and cannot be determined from retrospective data alone. The appropriate next step is a silent prospective evaluation—running the model in the background without acting on its predictions—to assess whether alert timing aligns with clinically observed deterioration events and to gather evidence on operational feasibility. Decision-threshold selection, selective prediction (abstention), and integration of additional signals can further reduce false positives under operational constraints. To examine whether the observed precision advantage in the single-split evaluation is attributable only to the default threshold, we conducted two complementary threshold optimization analyses. First, we identified the threshold that maximizes CE baseline precision and evaluated both methods at that threshold (Table A1). This analysis shows that the CE baseline can match the proposed method’s precision at a highly conservative operating point, but only with very few positive predictions and very low recall. Therefore, precision maximization alone does not define an operationally desirable threshold, and threshold-tuned precision must be interpreted together with recall and F1-score. Second, we compared precision at matched recall levels across four detection rates (10%, 20%, 30%, and 50%; Table A2). Together, these analyses suggest that the observed precision advantage in the single-split evaluation is not explained only by the default decision threshold. At the same time, because threshold selection changes the precision–recall balance and the matched-recall differences are small, these results should be interpreted as supplementary threshold analyses at the evaluated operating points rather than proving uniform superiority under every possible threshold setting.

### 6.2. Analysis of Why the Method Reduces False Positives

This subsection explains why the proposed method reduces false positives by first leveraging ablation evidence and then supporting the interpretation with visualization results. The pair-design ablation in Table 5 showed the roles of the pairwise terms in the contrastive objective. Single-pair variants indicated that TN–FP mainly supports false-positive suppression, whereas TP–FN mainly supports true-positive retention. Specifically, the TN–FP-only setting suppressed false positives to FP=55 but reduced true positives to TP=8, indicating a trade-off between false-positive suppression and detection retention. In contrast, the TP–FN-only setting retained TP=12 but left FP=135, indicating persistent confusion within the predicted-positive set. Therefore, the proposed method reduced false positives by shaping the same-label structure via TP–FN and TN–FP, while reducing TP/FP confusion within the predicted-positive set through a margin on TP–FP pairs.

This interpretation is supported by the UMAP analysis in Figure 6. In the baseline, TP and FP clustered into a shared region, whereas the proposed method spread TP and FP more broadly and reduced local entanglement. Overall, the proposed loss directly encouraged TP/FP separation within the predicted-positive set. This behavior supported its suitability for precision-oriented alerting.

A potential concern is whether the model simply copies the current-day health status rather than learning genuine predictive patterns. The “current-day health deterioration flag” records whether the current day is a deterioration day, is observable at prediction time, and was available to all models equally. On days with “current-day health deterioration” (flag = 1, n=252), a naive persistence rule that predicts all such days as positive achieves only a precision of approximately 34%, whereas the proposed method selectively predicted 10 of the 252 days as positive and achieved a precision of 70%. This result indicates that the model discriminates genuinely high-risk continuation days rather than simply copying the current label.

### 6.3. Impact of Loss Hyperparameters and Model Architecture

This subsection summarizes how architecture choices and loss hyperparameters affect performance and provides practical guidance for setting an operating point. Varying MLP width and depth yielded only limited improvements, indicating that model capacity alone did not substantially improve positive detection under severe class imbalance. The loss weight λ is the primary parameter that controls the trade-off between false-positive suppression and true-positive retention (Table 6). On this dataset, the F1-score peaked at λ=0.7 while precision peaked at λ=0.8, and λ∈[0.6,0.8] forms a practical search range, with λ=0.7 for F1-oriented operation and λ=0.8 for precision-oriented alerting. The margin *m* is a secondary parameter that adjusts TP–FP separation pressure within the predicted-positive set and fine-tunes the number of false positives (Table 7). On this dataset, m=0.3 yielded the most favorable balance between precision and F1-score, so a practical procedure first sets λ to choose an operating point and then tunes *m* to refine the margin effect.

### 6.4. Limitation and Future Work

This study clarified opportunities for improvement toward practical deployment. First, the best setting achieved a precision of 12.94%, corresponding to approximately 7 false alerts for every actual alert (74 false positives per 11 true positives in the test set). While this represents a meaningful improvement over the cross-entropy baseline, further reductions in false-alarm burden are needed before practical deployment. Combining the proposed loss with decision-threshold selection or selective prediction offers a concrete path toward this goal. Second, this study relied on data from a single care facility, providing a controlled proof of concept. A 5-fold cross-validation confirmed that the false-positive suppression effect was consistent across different data partitions within this facility (Table 4). However, because absolute precision varied substantially across folds, the precision estimate from this single-split evaluation should be generalized cautiously. Multi-facility validation presents a natural next step to assess whether the findings generalize across different recording practices, resident populations, and operational contexts. Third, the UMAP analysis revealed a region with concentrated false negatives, identifying positive patterns that current features and model capacity do not yet capture. This residual structure points to a concrete target for improvement: incorporating higher-frequency time series or event-level information could substantially increase true-positive detection. Fourth, the health deterioration label relies on keyword extraction from free-text care records, which may miss deterioration events recorded without the predefined terms. Integrating NLP-based label extraction or cross-referencing with formal incident records would strengthen label coverage and improve evaluation reliability.

Fifth, the hyperparameter sensitivity analysis (Table 6 and Table 7) shows non-monotonic behavior: for example, λ=0.5 performs worse than λ=0.4 despite the higher contrastive weight. This arises because the contrastive loss is non-convex and because the loss gradient at each iteration depends on mini-batch composition. Under severe class imbalance in the training split, 87.3% of mini-batches per epoch contained no positive samples, meaning the contrastive loss was computed in only 12.7% of batches. This makes contrastive updates infrequent and introduces variance into the optimization trajectory. Nevertheless, the 5-fold cross-validation results showed that the proposed method achieved the highest precision in all five folds, confirming that the false-positive suppression effect is robust to different data partitions despite this sensitivity. Future work can integrate operational design to control false-alarm burden. In addition, future work can combine decision-threshold selection with selective prediction (abstention) and alert prioritization to match operational requirements. Future work can also incorporate additional signals to better identify samples near the FN region, including higher-frequency time series and event-level information. The proposed loss is backbone-agnostic; it can therefore be combined with sequential architectures such as LSTMs or Transformers, which may better capture temporal dependencies in longitudinal care records. Applying the proposed loss to sequential encoders is a natural next step for further improving performance. Comparison with supervised contrastive learning methods designed for long-tailed recognition, such as SupCon [34] and PaCo [36], is also a valuable direction for future work. Those methods optimize for balanced class accuracy or F1 and may sacrifice precision under severe imbalance, whereas the proposed loss directly targets false-positive suppression. A direct comparison would clarify the complementary roles of outcome-based and label-based contrastive objectives in precision-oriented alerting tasks. Finally, external validation across facilities and time periods can assess deployment and robustness under varying operational conditions. Furthermore, adopting a positive-guaranteed sampling strategy that ensures at least one positive sample per mini-batch would increase contrastive loss updates and could improve stability under severe class imbalance.

## 7. Conclusions

In this paper, we proposed an outcome-based contrastive loss for imbalanced binary classification and applied it to alert to next-day health deterioration from care records and meteorological data. To reduce false positives and improve precision, we designed an outcome-based contrastive loss based on TP/FP/FN/TN outcomes. In particular, compared with the best baseline (random oversampling with standard cross-entropy), precision improved from 3.97% to 12.94% (+8.97 percentage points), while limiting the F1-score decrease to 0.71 percentage points (from 7.28% to 6.57%). Relative to the Model 3 class-weighted cross-entropy baseline used to isolate the loss effect, the proposed method reduced false positives from 135→74 while increasing true positives from 7→11. These results indicate that the proposed method can reduce unnecessary alerts while preserving useful positive detections, a step toward alert systems that staff can trust to detect deterioration earlier in understaffed care facilities.

The UMAP visualization of the representations showed stronger TP/FP separation under the proposed method than under the baseline. This pattern supports false-positive suppression from a representation-learning perspective. Furthermore, the pair-design ablation revealed distinct roles for different pair types, which strengthens this interpretation. Specifically, TN–FP pairs contributed to false-positive suppression, whereas TP–FN pairs contributed to true-positive retention. However, a region with a concentration of false negatives persists. This region suggests positive patterns that are not captured by the current features or model capacity. Therefore, future work can incorporate additional signals and operational design. For example, future work could employ decision-threshold selection and selective prediction to balance the false-alarm burden and detection performance. In addition, external validation across facilities and time periods can evaluate generalization and robustness under varying operational conditions.

## Figures and Tables

**Table 1:** Features computed from care-record data. Vital signs such as body temperature and blood pressure can be measured multiple times per day, so daily averages are used. The health deterioration flag and counts reflect the resident’s health history up to and including the current day, all of which are observable at prediction time.

Data	Calculation
Medication Rehabilitation and recreation Oral care Bathing and cleansing Snack Outing support Family/visitor visits Dressing and bed mobility Transportation support	Count
Nurse call	
Main dish intake Side dish intake Water intake Meal assistance level Amount of excretion Amount of urination	Mean Sum Difference in mean from the previous day Difference in sum from the previous day
Body temperature SpO2 Heart rate Systolic blood pressure Diastolic blood pressure	Mean
Average temperature Precipitation Sunshine hours Average wind speed Maximum temperature Minimum temperature Maximum wind speed	Value Difference from the previous day
Health deterioration flag (current day) Health deterioration count (past 7 days) Health deterioration count (past 14 days) Health deterioration count (past 21 days)	Flag (0/1) Count

**Table 2:** Baseline prediction performance of each model configuration using only class-weighted cross-entropy loss ($L_{\mathrm{CE}(\mathrm{we})}$).

Model	Unit	Depth	TP	FN	FP	TN	F1 (%)	Prec (%)
1	16	3	12	238	469	22,023	3.28	2.49
2	16	4	12	238	552	21,940	2.95	2.13
3	32	5	7	243	135	22,357	3.57	4.93
4	32	6	6	244	93	22,399	3.44	6.06

**Table 3:** Performance comparison between baseline methods and the proposed method (λ=0.7, m=0.3). Random upsampling and SMOTE are compared in training with standard cross-entropy and class-weighted cross-entropy losses.

Model	Loss	TP	FN	FP	TN	F1 (%)	Prec (%)
3	RS, $L_{CE(normal)}$	110	140	2660	19,832	**7.28**	3.97
3	RS, $L_{CE}$	224	26	16,778	5714	2.60	1.33
3	SMOTE, $L_{CE(normal)}$	247	3	21,768	724	2.22	1.12
3	SMOTE, $L_{CE}$	247	3	21,747	745	2.22	1.12
3	$L_{FocalLoss}$	160	90	7952	14,540	3.83	1.97
3	$0.3L_{CE}+0.7L_{cont}$	11	239	74	22,418	6.57	**12.94**

**Table 4:** 5-fold cross-validation results comparing TP, FN, FP, TN, F1, and precision for baseline methods and the proposed method (λ=0.7, m=0.3). Values denote mean ± standard deviation across folds.

Loss	TP	FN	FP	TN	F1 (%)	Prec (%)
RS, $L_{CE(normal)}$	46.0±44.7	115.0±44.8	1278.4±175.4	16,346.2±225.6	5.57±4.69	3.23±2.86
RS, $L_{CE}$	138.4±80.9	22.6±12.0	11,412.0±800.2	6212.6±830.7	2.32±1.23	1.18±0.63
SMOTE, $L_{CE(normal)}$	18.0±18.8	143.0±68.8	332.8±112.0	17,291.8±111.3	6.19±4.56	5.28±5.04
SMOTE, $L_{CE}$	126.4±72.3	34.6±18.1	10,152.0±647.5	7472.6±643.2	2.41±1.32	1.23±0.68
$L_{FocalLoss}$	77.0±56.3	84.0±31.6	2328.8±225.6	15,295.8±271.6	5.69±3.46	3.09±1.94
$0.3L_{CE}+0.7L_{cont}$	45.4±48.8	115.6±39.8	319.2±97.4	17,305.4±177.3	12.87±10.48	9.47±8.09

**Table 5:** Performance comparison across all $2^{3}-1=7$ non-empty subsets of the three main pair types (TP–FN, TN–FP, TP–FP)—including the full proposed method as the three-term subset—together with a negative-control variant (FN–TN), under a fixed setting (Model 3, λ=0.7, m=0.3).

Model	Loss	TP	FN	FP	TN	F1 (%)	Prec (%)
3	$0.3L_{CE}+0.7L_{cont(\mathrm{TP}\text{-}\mathrm{FN})}$	12	238	135	22,357	6.05	8.16
3	$0.3L_{CE}+0.7L_{cont(\mathrm{TN}\text{-}\mathrm{FP})}$	8	242	55	22,437	5.11	12.70
3	$0.3L_{CE}+0.7L_{cont(\mathrm{TP}\text{-}\mathrm{FP})}$	9	241	142	22,350	4.49	5.96
3	$0.3L_{CE}+0.7L_{cont(\mathrm{FN}\text{-}\mathrm{TN})}$	0	250	2	22,490	0.00	0.00
3	$0.3L_{CE}+0.7L_{cont(\mathrm{TP}\text{-}\mathrm{FN},\mathrm{TN}\text{-}\mathrm{FP})}$	8	242	182	22,310	3.64	4.21
3	$0.3L_{CE}+0.7L_{cont(\mathrm{TP}\text{-}\mathrm{FN},\mathrm{TP}\text{-}\mathrm{FP})}$	12	238	84	22,408	6.94	12.50
3	$0.3L_{CE}+0.7L_{cont(\mathrm{TN}\text{-}\mathrm{FP},\mathrm{TP}\text{-}\mathrm{FP})}$	10	240	93	22,399	5.67	9.71
3	$0.3L_{CE}+0.7L_{cont}$ (proposed)	11	239	**74**	22,418	6.57	**12.94**

**Table 6:** Performance comparison across loss weight λ in the proposed method (m=0.3). All results were obtained by training Model 3 using $L_{\mathrm{total}}=\left(1-\lambda \right)L_{\mathrm{CE}}+\lambda L_{\mathrm{cont}}$.

Model	λ	TP	FN	FP	TN	F1 (%)	Prec (%)
3	0.1	10	240	216	22,276	4.20	4.42
3	0.2	10	240	248	22,244	3.94	3.88
3	0.3	8	242	117	22,375	4.27	6.40
3	0.4	10	240	261	22,231	3.84	3.69
3	0.5	9	241	313	22,179	3.15	2.80
3	0.6	10	240	111	22,381	5.39	8.26
3	0.7	11	239	74	22,418	**6.57**	12.94
3	0.8	7	243	36	22,456	4.78	**16.28**
3	0.9	5	245	209	22,283	2.16	2.34

**Table 7:** Performance comparison across margin *m* in the proposed method (fixed Model 3, λ=0.7).

Model	*m*	TP	FN	FP	TN	F1 (%)	Prec (%)
3	0.1	11	239	160	22,332	5.23	6.43
3	0.3	11	239	74	22,418	**6.57**	**12.94**
3	0.5	9	241	71	22,421	5.45	11.25
3	0.7	11	239	124	22,368	5.71	8.15
3	0.9	11	239	101	22,391	6.08	9.82
3	1.0	9	241	67	22,425	5.52	11.84
3	1.1	9	241	70	22,422	5.47	11.39
3	1.3	11	239	82	22,410	6.41	11.83

## Data Availability

Restrictions apply to the availability of the care-record data. The care-record data were obtained from AUTOCARE LLC and are not publicly available because the authors do not have permission to redistribute them. Requests to access these data should be directed to the corresponding author and are subject to permission from the data owner. The meteorological data are publicly available from the Japan Meteorological Agency [39].

## Appendix A. Precision-Recall Curves

Figure A1 shows the precision-recall curves for the proposed method (λ=0.7, m=0.3) and the CE baseline on the single-split test set. The proposed method achieved AUPRC =0.045, compared with AUPRC =0.032 for the CE baseline. The dotted line represents the expected performance of a random classifier at the observed class prevalence (=0.011). The higher AUPRC of the proposed method indicates better overall precision–recall performance on the single-split test set, suggesting that the observed precision advantage is not limited to the default threshold of 0.5.

## Appendix B. Threshold Robustness Analysis

The following analyses use the same single-split test set as Table 3 and Figure A1.

### Appendix B.1. Method A: Performance at CE Baseline’s Precision-Maximized Threshold

To examine the CE baseline under a precision-maximized operating point, we identified the threshold that maximizes CE baseline precision, then evaluated both methods at that shared threshold. Because this threshold yields very few positive predictions, this analysis should be interpreted as a supplementary check of a highly conservative CE-baseline operating point rather than as an operationally preferred threshold. This result indicates that maximizing precision alone can produce a nearly non-alerting operating point, so threshold-tuned precision must be interpreted together with recall and F1-score.

**Table A1:** Confusion-matrix counts and performance metrics when both methods operate at CE baseline’s precision-maximized threshold. The CE baseline achieves maximum precision (33.33%) at threshold 0.89; the proposed method is evaluated at the same threshold.

Method	Threshold	TP	FN	FP	TN	Precision (%)	Recall (%)	F1-Score (%)
CE Baseline	0.89	3	247	6	22,486	33.33	1.20	2.32
Proposed	0.89	2	248	4	22,488	33.33	0.80	1.56

### Appendix B.2. Method B: Precision at Matched Recall Levels

To further examine whether the observed precision advantage depends on a specific decision threshold, we evaluated both methods when constrained to the same recall (detection) level. At each of the four target recall levels (10%, 20%, 30%, and 50%), we identified the threshold for each method that achieved or closely matched that recall, then compared their corresponding precision values. Table A2 shows that the proposed method achieved slightly higher precision than the CE baseline at all four tested recall levels, although the differences were small (0.14–0.95 percentage points). These results suggest that the observed precision advantage is not explained only by the default threshold.

**Table A2:** Precision comparison at matched recall levels. For each target recall level, the threshold for both methods was set to achieve approximately that recall. The proposed method achieved slightly higher precision than the CE baseline across the evaluated operating points, although the differences were small.

Target	CE Baseline			Proposed Method			Precision
Recall	Threshold	Precision	Actual Recall	Threshold	Precision	Actual Recall	Difference
(%)		(%)	(%)		(%)	(%)	(pp)
10	0.322	2.56	10.00	0.338	3.51	10.00	+0.95
20	0.309	3.83	20.00	0.314	4.09	20.00	+0.26
30	0.296	4.22	30.00	0.301	4.50	30.00	+0.29
50	0.222	2.67	50.00	0.238	2.81	50.00	+0.14
